# Bats and Academics: How Do Scientists Perceive Their Object of Study?

**DOI:** 10.1371/journal.pone.0165969

**Published:** 2016-11-10

**Authors:** Christophe Boëte, Serge Morand

**Affiliations:** 1 UMR Emergence des Pathologies Virales, Aix Marseille Université, IRD 190, EHESP, INSERM 1207, 27, Bd Jean Moulin 13385, Sarseille, Cedex 5, France; 2 CNRS ISEM-CIRAD AGIRs, Centre d’Infectiologie Christophe Mérieux du Laos, Vientiane, Lao PDR; 3 Department of Helminthology, Faculty of Tropical Medicine, Mahidol University, Bangkok, Thailand; University of Western Ontario, CANADA

## Abstract

Bats are associated with conflicting perceptions among humans, ranging from affection to disgust. If these attitudes can be associated with various factors among the general public (e.g. social norms, lack of knowledge), it is also important to understand the attitude of scientists who study bats. Such reflexive information on the researchers community itself could indeed help designing adequate mixed communication tools aimed at protecting bats and their ecosystems, as well as humans living in their vicinity that could be exposed to their pathogens. Thus, we conducted an online survey targeting researchers who spend a part of their research activity studying bats. Our aim was to determine (1) how they perceive their object of study, (2) how they perceive the representation of bats in the media and by the general population, (3) how they protect themselves against pathogen infections during their research practices, and (4) their perceptions of the causes underlying the decline in bat populations worldwide. From the 587 completed responses (response rate of 28%) having a worldwide distribution, the heterogeneity of the scientists’ perception of their own object of study was highlighted. In the majority of cases, this depended on the type of research they conducted (i.e. laboratory versus field studies) as well as their research speciality. Our study revealed a high level of personal protection equipment being utilised against pathogens during scientific practices, although the role bats play as reservoirs for a number of emerging pathogens remains poorly known. Our results also disclosed the unanimity among specialists in attributing a direct role for humans in the global decline of bat populations, mainly via environmental change, deforestation, and agriculture intensification. Overall, the present study suggests the need for better communication regarding bats and their biology, their role within the scientific community, as well as in the general public population. As a consequence, increased knowledge regarding scientists’ perceptions of bats should improve the role scientists play in influencing the perception of bats by the general public.

## Introduction

The perception of the general public regarding bats can be quite ambivalent, i.e. from the positive association with a brave and tormented superhero lacking superpowers who fights crime and protects the fictional Gotham City, the indomitable Batman [1], to the recent negative link with the emergence of diseases such as Ebola and Nipah. A clear example of a perceived danger associated with bats is the theme of the movie *Contagion* by S. Soderbergh, in which the chain of contagion of a deadly virus in the human population starts from bats and pigs after the disturbance of a bat colony due to deforestation [2]. If these conflicting perceptions are widespread among the general public who do not have close contact with bats, we aimed to investigate the perception of scientists studying bats who have greater knowledge and frequent interactions with them. Basically, this addresses the fact that knowledge on these aspects can facilitate rational evaluation and analysis of risks to public health. As a consequence this should provide scientists and science communicators adequate material to modify the norms shaping the publics' views on bats. We specifically aimed to explore the perception of bats among scientists in relation to their potential roles as reservoir hosts of zoonotic diseases. Bats are often associated with the emergence of pathogens [3, 4], which has created a negative impact in the public. However, it is expected that knowledge dispels fear in the scientific community or that several research experts view bats only in terms of infection, risk, and danger. Thus, the presentation of bats varies widely among scientists, as epidemiologists may choose frightening pictures of bats [5], while biological conservationists may choose appealing ones [6]. Obviously, this remains highly subjective. Through questioning scientists who mainly study bats, our aim was to determine (1) how scientists perceive their object of study, (2) how they perceive the representation of bats in the media and by the general public, (3) how they protect themselves and others against pathogen infections in their research practices, and (4) the causes that may explain the ongoing decline in bat populations worldwide. Overall the results of this study should help creating integrated educational programmes aimed at protecting bats and their ecosystems, as well as humans living in their vicinity. This requires an approach that is able to deliver a mixed message to conciliate issues related to bat conservation and ecosystem protection, while ensuring proper information on infectious risk transmission and thus public health.

## Material and Methods

A 29-question survey was designed, with most questions related to the location, nationality, type of research, research practices, the researcher’s own perception of bats, as well as their views on the general public perception on bats. We also included a quiz to test the researcher’s knowledge regarding bats and the role of bats as reservoir hosts of pathogens, as well as a series of questions related to the causes of the global decline in bat populations. These detailed questions and responses are available in the S1 File.

The questionnaire was sent to the corresponding authors of one or more scientific papers published between 2010 and 2014 that were referenced in the database ISI Web of Science as related to Chiroptera. The choice was based on the occurrence of a series of combined keywords (see S2 File). References were then checked to avoid the selection of unrelated publications. Once the duplicates were removed, the questionnaire was sent out to valid email addresses. When the same email address was found for several publications in different years, the most recent one was retained and the others were discarded. The questionnaire was sent through the online survey platform QuestionPro (http://www.questionpro.com/) to 2,180 potential participants in May 2015 and was closed in June 2015 after three reminders had been sent. Statistical analyses were performed using R software [7] and model selection was performed with the use of the package glmulti [8]. A total of 610 completed responses were received, accounting for an average response rate of 27.98%. We obtained the following increasing response rates from 2010 to 2014: 17.8% (51/287), 22.8% (88/386), 24.9% (96/386), 29.6% (170/574), and 37.5% (205/546). From the total responses, 23 were from researchers who declared they were not working on bats, with 587 responses remaining that contained usable answers for further analysis.

The survey was performed in compliance with the Helsinki Declaration [9], i.e. all participants were informed about the aim of the questionnaire and were free not to participate or to withdraw at any stage of the process. Responses were analysed in an anonymous manner.

## Results

### A worldwide distribution

As illustrated in S1 Fig, respondents showed a worldwide distribution in their research activities on bats, with Eurasian (18.6%) and North American (16.1%) regions being the most represented.

### Type of research

**Fig 1:**
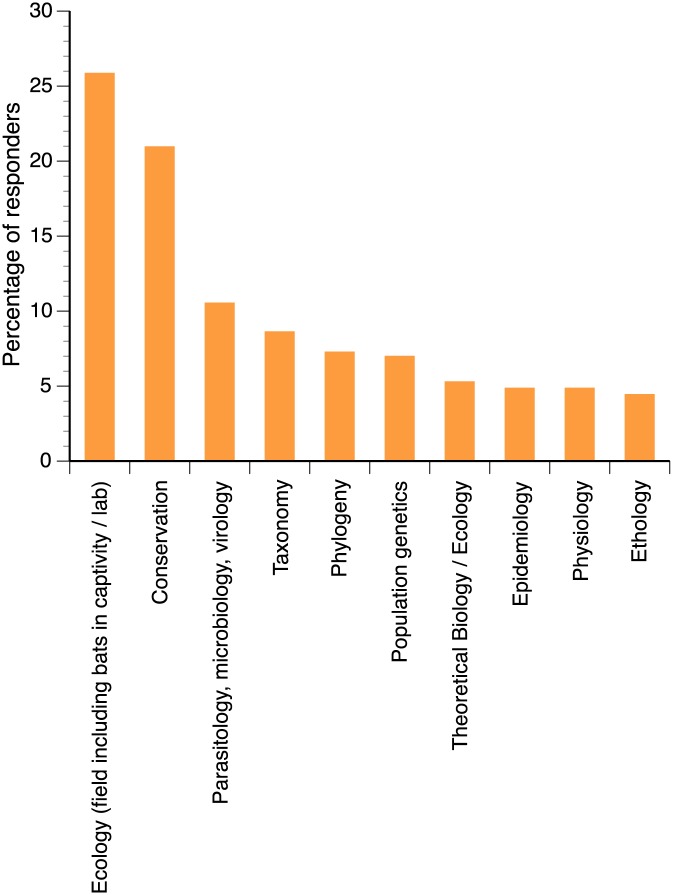
Distribution of the respondents according to their major topic of research (n = 587).

Research on bats represented an average of 55.7% ± 1.4 of the research activities of our studied population, with these activities being divided as follows: field activities (30.0% ± 1.0), laboratory studies (19.4% ± 0.9), and desk activities (51.7% ± 1.1) (Q2 and Q21). The most extensively studied bat family was Vespertilionidae, followed by Molossidae (Q5) (S2a Fig), with insects and invertebrates dominating the diets of these bats (S2b Fig). Slightly greater than one-third of the respondents studied only one family and the distribution of family numbers was positively skewed (S3 Fig) with a median number of 2. The main topics of research were field ecology, including bats in captivity or in the laboratory, and conservation biology (Fig 1) with 28% (164/587) of the respondents declaring that they studied only one topic, with the median number of research topics being 2.

### Perception of bats

Although 96.3% of the scientists did not perceive bats as dangerous animals, several factors can lead to differences in this perception, i.e. studying the physiology of bats as well as bat infections were the likely reasons why bats were considered dangerous animals by the responders whereas this is the opposite when studying their ecology (Table 1).

**Table 1:** Logistic regression of the perception of bats as dangerous animals by researchers (A higher number of asterisks * describes a higher level of statistical significance of the associated factor included in the analysis).

	Estimate	Standard error	z value	Pr (>lzl)
(Intercept)	–3.811	0.529	–7.199	6.11e-13 ***
Ecology	–2.67	0.786	–3.4	0.000662 ***
Epidemiology	–0.946	0.716	–1.322	0.186
Physiology	1.786	0.641	2.784	0.005367 **
Working on bat infection	2.121	0.615	3.452	0.000556 ***

The trend differed when scientists were asked about their views on the general public perceptions of bats, as a majority of the respondents (70.8%) believe that lay-people perceive bats as dangerous animals. Statistical analysis reveals that several factors might explain this perception (Table 2). Thus, scientists involved in laboratory-orientated research are more likely to share this public perception, whereas those studying ethology, conservation, or bat infections are less likely to share the perception that bats are dangerous animals.

**Table 2:** Logistic regression of the representation of bats as a dangerous animal by lay-people according to scientists studying bats. (A higher number of asterisks * describes a higher level of statistical significance of the associated factor included in the analysis).

	Estimate	Standard error	z value	Pr (>lzl)
(Intercept)	0.479	0.286	1.673	0.09423
Conservation	–0.481	0.245	–1.961	0.04991 *
Ecology	0.375	0.253	1.478	0.13951
Theoretical research	0.54	0.342	1,581	0.11391
Ethology	–0.693	0.324	–2.140	0.03238 *
Working on bat infection	–0.653	0.232	–2.820	0.00480 **
Percentage of research on bats	0.006	0.003	1.656	0.09782
Percentage of laboratory research	0.018	0.006	3.097	0.00196 **
Manipulation of live bats	0.394	0.232	1.702	0.08871

**Fig 2:**
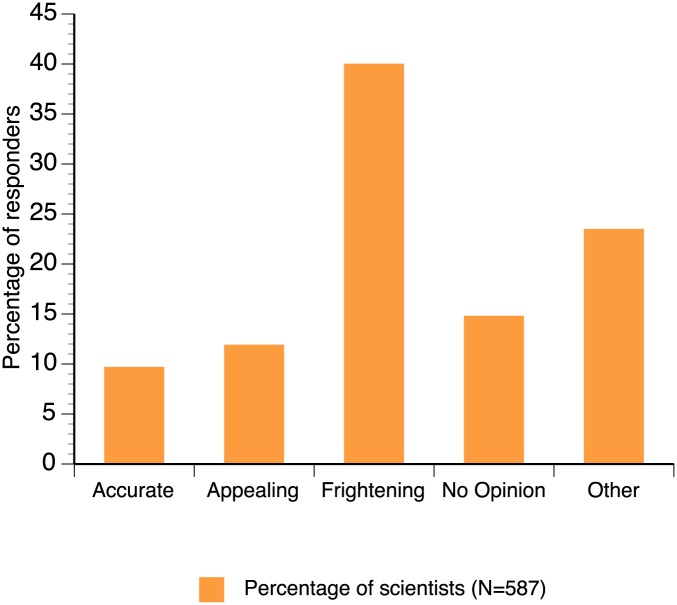
Representation of the percentage of respondents according to their perception of the portrayal of bats in the media (n = 587).

Similarly, 36.8% of the laboratory-oriented scientists considered that the general population preferred bats over rats, while 40.9% of the field-oriented scientists believed that the general population considered bats similar to rats. Such differences may be explained by the negative representation of bats in the media (Fig 2). A large proportion of scientists (approximately 40%) declared that bats are presented as frightening in the media; however, scientists who chose ‘Other’ (nearly 25%) as their response declared that the manner in which bats are portrayed in the media is inaccurate, misleading, and varying.

With regard to the consumption of bats, only 10 respondents in our survey declared that they had consumed bats, although it did not constitute a food habit. The opinions about the consumption of bats were quite diverse and appeared to rely on respondents’ views on bat conservation as well as on culinary taste “*De gustibus non est disputandum*” [10].

### Bats and pathogens

**Fig 3:**
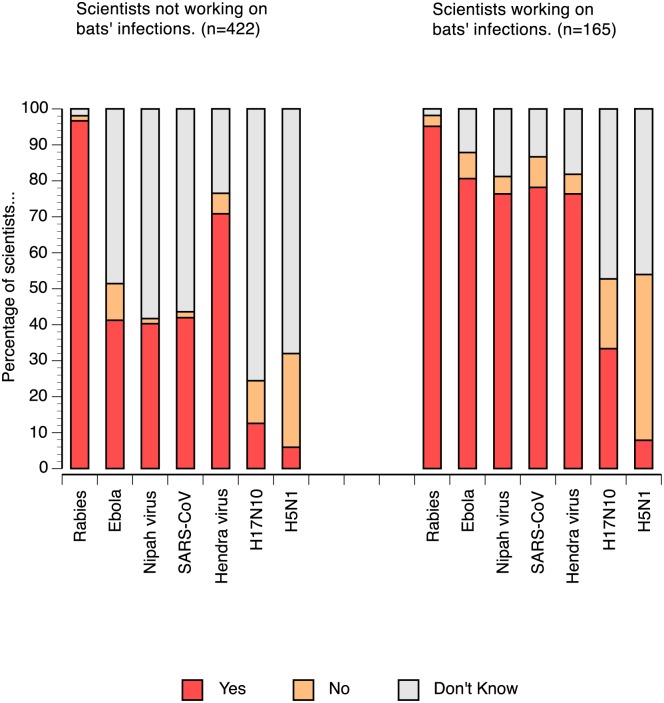
Level of information of respondents regarding the role of bats as reservoir hosts for several pathogens (n = 587). The following groups 'Yes', 'No' and 'Don't know' correspond respectively to an agreement with the statement that bats are reservoir of the given pathogen, a disagreement with this statement and the absence of knowledge on the topic respectively.

Scientists who do not study bat infections disagree with the putative role of bats as reservoir hosts for pathogens presented in the quiz, except for rabies and, to a lesser extent, the Hendra virus. However, knowledge about bats being potential reservoir hosts for rabies and the Hendra virus was equally shared and highly widespread among the respondents (Fig 3). We introduced the avian flu H5N1 in our quiz on bats as potential reservoir hosts of pathogens and few scientists responded incorrectly by classifying it as potential virus carried by bats. A large proportion of scientists (46%) working on infection in bats disagree that bats were a reservoir of H5N1. A large fraction of the respondents in both groups (47.3% of the group studying infection and 75.6% of the other group) declared that they disagreed that bats can be reservoir hosts of the bat influenza virus H17N10 [11]. In addition, 48.6% to 58.3% of the researchers studying bats (but not their infections) disagreed that bats could be reservoir hosts of viruses such as Ebola, Nipah, and SARS-CoV.

**Fig 4:**
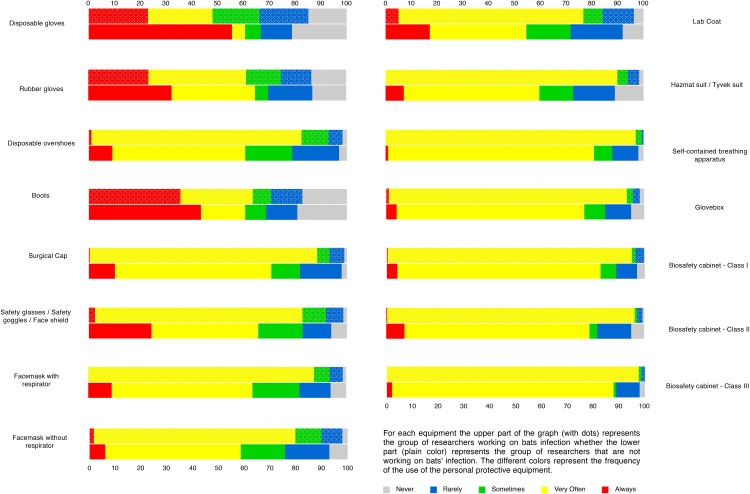
Percentage of respondents manipulating live bats using personal protective equipment. The total number of scientists corresponds to the number of scientists (n1 = 395) manipulating live bats, and is therefore a subset of the 587 respondents.

**Fig 5:**
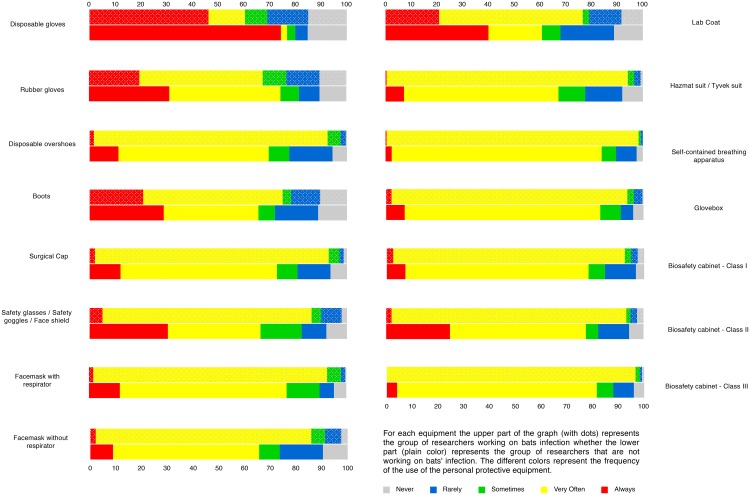
Percentage of respondents manipulating samples removed from live bats using personal protective equipment. The total number of scientists (n2 = 365) corresponds to the number of scientists manipulating live bats, and it is therefore a subset of the 587 respondents.

We also questioned scientists about the use of personal protective equipment while manipulating live bats as well as while collecting samples from live bats. When manipulating live bats, a majority of scientists responded that they always or very often wore personal protective equipment (Fig 4). Researchers studying bat infection always used personal protective equipment, especially disposable gloves, safety goggles, safety glasses or face shields when manipulating live bats. A similar trend was observed among researchers manipulating samples collected from live bats (Fig 5). Multiple correspondence analysis on the use of personal protective equipment for the manipulation of live bats as well as while collecting samples from live bats revealed that the frequency of using one type of personal protective equipment was highly predictive of the use of another type, especially when studying bat infections. In other words, if the researcher regularly used disposable overshoes, then they also frequently used a surgical cap (S4 File) during their research activities.

### Bats, conservation, and biodiversity

**Fig 6:**
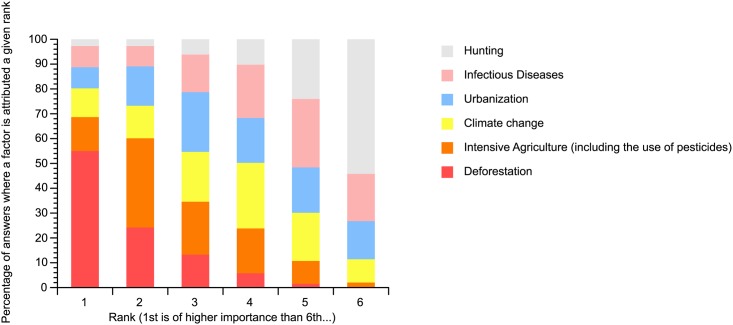
Rank of importance of the different factors explaining the worldwide decline in bat populations according to respondents (n = 587).

Worldwide bat populations are declining [12, 13] due to several factors [14]. According to the respondents of our survey, deforestation and intensive agriculture are the major detrimental factors followed by urbanisation and climate change (Fig 6).

We conducted logistic regression on the ranking of these factors by respondents (S3 File). Deforestation ranked high on the list of causes responsible for the decline of bat populations appears clearly related to working directly with live bats or samples taken from live bats. Similarly, high ranking of intensive agriculture is related to a low percentage of laboratory activities, manipulation of samples, and research activities in the field of conservation. Surprisingly, studying live bats appears to be related to low ranking of intensive agriculture; however, this was likely due to an association with deforestation and intensive agriculture being ranked as the major factors of the global decline of bats.

## Discussion

Overall, our survey reveals several important aspects. First, it appears that the idea of the danger associated with bats is not uniform within the scientific community studying them. Scientists can be classified into two categories: laboratory-oriented versus field-oriented, or as epidemiologists versus non-epidemiologists. The perceptions of scientists on bats appear to clearly depend on their main research activities and their contact with bats, and their internal tissues and/or blood, either in the laboratory or in the wild.

The idea behind the danger associated with bats might be related to the relative lack of information regarding several diseases transmitted through bats. Thus, it is important to improve communication amongst scientists and to circulate accurate information within the scientific community. There are numerous popular misconceptions associated with bats [15]. Thus, bats are often affiliated with self-reported phobias, as are snakes and mice [16], and they are highly ranked among the animals for which people have the feeling of disgust [17, 18]. Bats are also associated with vampires and blood drinking, as was shown in a survey in Slovakia that revealed that 20% of the college students believed that most bats drink blood, while in reality only three species do [19]. This lack of knowledge is particularly crucial when explaining the fear or reluctance people might have towards bats; therefore, better knowledge on the real level of infectious risks associated with bats would be helpful, especially for any conservation approach [20, 21]. If this is valid in professionals working closely with bats, accurate information on bat biology might also help the public to better understand the biology of bats and their roles in ecosystems. Such a trend has already been observed among students in Slovakia, where increased knowledge about bats is positively associated with a positive perspective [19] and improvement in the perception of bats is having a positive impact on efforts towards their conservation, at least in a population that has an interest in bats [15]. This would also be valid for any programme aimed at protecting wildlife that is often portrayed as the source of emerging infectious diseases [22].

Conflicting views exist on the management of bat populations in terms of conservation and public health. As mentioned previously, by investigating the perception of bats by scientific experts, our survey highlights heterogeneity regarding the perception of bats among a well-informed community of specialists. These results should be compared with other surveys conducted on local communities and stakeholders. It is essential to elucidate how bats are perceived in order to develop adequate communication tools aimed at protecting bats and their ecosystems, as well as humans living in their vicinity. This requires an approach that is able to deliver a mixed message to conciliate issues related to bat conservation and ecosystem protection, while ensuring proper information on infectious risk transmission and thus public health are also stated [21].

The association between bats and the danger of pathogen transmission often exists and our study clearly reveals the widespread use of personal protective equipment against infection, especially by scientists studying infection in bats. This is a positive aspect in terms of work safety as well as for public health in order to avoid the spread of an eventual bat pathogen in the human population. Apart from the obvious advantages that the use of adequate protection by scientists facilitates the effective manipulation of bats; however, the white nose syndrome in bats caused by *Pseudogymnoascus destructans* can also be transmitted by humans through their clothes and gear as they move from one cave to another [23, 24]. Given the fact that 20% of the scientists in our survey have declared that they are currently studying *P*. *destructans*, it is important that these scientists should take precautions to not transmit infections among themselves or in the bat colonies they are studying.

Our study clearly reveals that scientists consider that environmental modifications by humans—direct, such as deforestation and intensive agriculture, or indirect, such as climate change—are the major factors causing worldwide decline in bat population [25, 26]. This clearly highlights the human role on a worldwide scale and emphasises the need for ecosystem protection in conservation programmes.

Finally, a large majority of scientists associate the general population as having a negative perception about bats. Based on this fact, it would also be highly informative to measure the level of public engagement of the respondents of our survey (e.g. interacting with a non-scientific audience, show-casing the relevance of their research, creating awareness, etc.), and the impact it could have on supporting measures that favour the conservation of bat populations.

## Supporting Information

S1 Fig Repartition of responders according to their place of work and research activities on bats (n = 587). (EPS)

S2 Fig **a)** Repartition of the responders according to the families of bats they are working on (n = 587). **b)** Fig Repartition of the responders according to the type of food of the bats they are working on. (n = 587).
(EPS)

S3 Fig Repartition of the responders according to the number of families of bats they are working on. (n = 587).
(EPS)

S1 File Invitation, text of the survey and notes. (RTF)

S2 File Combination of keywords used to select participants in the survey. (RTF)

S3 File Statistical analysis of the factors explaining the cause of the global decline of bats according to responders. (RTF)

S4 File Analysis on the use of protection tools while manipulating alive bats or samples of alive bats. (DOC)
