# Supplementary material for: Bats and Academics: How Do Scientists Perceive Their Object of Study?
Source: PLoS One. 2016 Nov 10;11(11):e0165969. doi: 10.1371/journal.pone.0165969 (PMC5104368; doi:10.1371/journal.pone.0165969)
Supplement: S1 File — (RTF) [file pone.0165969.s004.rtf]

S1 File. Invitation, text of the survey and notes. 

Notes:

(1)

We thank Pr Andrew Cunningham for bringing our attention to the fact that we have not offered the choice of the use of leather gauntlets or other protective gloves (e.g. Chain mail gloves, venom defender gloves). 

(2) 
Apart from the first invitation sent on March, 17th to take part in the survey, we have been sending several reminders to non responders at the following days : March, 31st; April, 15th; March


Text of invitation

Dear colleague,

We are currently conducting a survey on the perception of bats by scientists studying them and we would value your opinion. We have been designing a short questionnaire "How scientists working on bats perceive their object of study?" and we would really appreciate if you could fill it as you have been the corresponding author of paper dealing with bats in the past years. If this is not the case, we are sorry for disturbing you but we would appreciate if you could start the questionnaire and answer the first question. That would be helpful for us and it would also prevent you from receiving other unwanted messages from us. If you are not interested by the survey you can unsubscribe from further correspondance by clicking on the 'Unsubscribe' link at the end of this message or drop us a line and we will remove your email address from our mailing-list.
Replying to the questionnaire is not going to be very time-consuming (It should take less than 10 mn to fill and most likely about 5 mn). If you receive our invitation more than once, we are sorry for disturbance and we kindly ask you to fill the survey only once. Thank you very much for your time and support! Feel free to contact us if you need additional information.
Please start with the survey now by clicking on the button below.

					START SURVEY


Q1. Is your research somehow related to bats?
	1. Yes 
	2. No

Test If answer to Q1 is YES, then Q2. If answer is No, then the responder is thanked and the questionnaire is finished.

Q2. Please allocate 100 points on how you spend your research activities:
	Research dealing with bats __________
	Research NOT dealing with bats __________

Q3. In which zoogeographic region * do you live and work most of the year?* based on an update of Wallace's Zoogeographic Regions of the World by Ben G. Holt et al. Science 339, 74 (2013). DOI: 10.1126/science.1228282 
1.	African
2.	Amazonian
3.	Arctico-Siberian
4.	Australian
5.	Chinese
6.	Eurasian
7.	Guineo-Congolian
8.	Indo-Malayan
9.	Japanese
10.	Madagascan
11.	Mexican
12.	North American
13.	Novozelandic
14.	Oriental
15.	Panamanian
16.	Papua-Melanesian
17.	Polynesian
18.	Saharo-Arabian
19.	South American 
20.	Tibetan

Q4. In which country or territory do you live and work most of the year?

Q5. In which zoogeographic regions(s) are you conducting most of your research activities on bats?
1.	African
2.	Amazonian
3.	Arctico-Siberian
4.	Australian
5.	Chinese
6.	Eurasian
7.	Guineo-Congolian
8.	Indo-Malayan
9.	Japanese
10.	Madagascan
11.	Mexican
12.	North American
13.	Novozelandic
14.	Oriental
15.	Panamanian
16.	Papua-Melanesian
17.	Polynesian
18.	Saharo-Arabian
19.	South American 
20.	Tibetan

Q6. On which family (or families) of bats are you working?
1.	Craseonoycteridae
2.	Emballonuridae
3.	Furipteridae
4.	Hipposideridae
5.	Megadermatidae
6.	Miniopteridae
7.	Molossidae
8.	Mormoopidae
9.	Mystacinidae
10.	Myzopodidae
11.	Natalidae
12.	Noctilionidae
13.	Nycteridae
14.	Phyllostomidae
15.	Pteropodidae
16.	Rhinolophidae
17.	Rhinopomatidae
18.	Thyropteridae
19.	Vespertilionidae

Q7. Do you work on bats that feed on:
1.	Fruits
2.	Nectar
3.	Insects and invertebrates
4.	Vertebrates
5.	Blood


Q8. What type of studies do you conduct on bats: 
1.	Conservation
2.	Ecology (field including bats in captivity / lab)
3.	Theoretical Biology / Ecology
4.	Ethology
5.	Epidemiology
6.	Parasitology, microbiology, virology
7.	Phylogeny
8.	Physiology
9.	Population genetics
10.	Taxonomy

Q9. Do you work on bats infections? 
1.	Yes
2.	No


Q10. Which infection(s) of bats are you working on? (please name the aetiological agents as precisely as possible and separate them with semicolon)

Q11. Could you please tick the correct answers:Bats can be reservoirs for the following pathogens:

	Yes	No 	Don't Know	
Rabies				
SARS-CoV				
Hendra virus				
Nipah virus				
Ebola				
H17N10				
H5N1				


Q12. How do you perceive the representation of bats in the media? 
1.	Accurate
2.	Accurate
3.	Frightening
4.	No opinion
5.	Other

Q13. Do you eat bat meat?
1.	Yes
2.	No

Q14. What do you think about the consumption of bat meat?

Q15. Do you, in general, perceive bats as dangerous animals?
1.	Yes
2.	No 
3.	No opinion

Q16. Do you think most people perceive that bats are dangerous animals?
1.	Yes
2.	No 
3.	Dont know

Q17. Do you think people prefer bats rather than rats?
1.	Yes
2.	No
3.	Similar
4.	Dont know

Q18. Do you manipulate alive bats?
1.	Yes
2.	No

Q19. While manipulating alive bats, do you use any of the following protection tools?

	Always	Very often	Sometimes	Rarely	Never	
Disposable gloves						
Rubber gloves						
Disposable overshoes						
Boots						
Surgical cap						
Safety glasses / Safety goggles/ Face shield						
Facemask with respirator						
Facemask without respirator						
Lab coat						
Hazmat suit / Tyvek suit						
Self-contained breathing apparatus						
Glovebox						
Biosafety cabinet - Class I						
Biosafety cabinet - Class II						
Biosafety cabinet - Class III						


Q20. Do you manipulate samples from alive bats?
1.	Yes
2.	No


Q21. While manipulating samples from alive bats, do you use any of the following protection tools?

	Always	Very often	Sometimes	Rarely	Never	
Disposable gloves						
Rubber gloves						
Disposable overshoes						
Boots						
Surgical cap						
Safety glasses / Safety goggles/ Face shield						
Facemask with respirator						
Facemask without respirator						
Lab coat						
Hazmat suit / Tyvek suit						
Self-contained breathing apparatus						
Glovebox						
Biosafety cabinet - Class I						
Biosafety cabinet - Class II						
Biosafety cabinet - Class III						


Q22. Please allocate 100 points on how you spend your working time on bats or on sample of bats:
1.	Field activities __________
2.	Lab activities __________
3.	Desk activities (data analysis, grant and paper writing...) __________


Q23. Considering the most important factors affecting the populations of bats worldwide, please drag the answers and rank then (1st to 6th) in order of importance:
1.	Climate change __________
2.	Deforestation __________
3.	Infectious Diseases __________
4.	Intensive Agriculture (including the use of pesticides) __________
5.	 Hunting __________
6.	 Urbanization __________


Q24. Which country or territory are you a national from?

Q25. Which zoogeographic region* are you national from? * based on an update of Wallace's Zoogeographic Regions of the World by Ben G. Holt et al. Science 339, 74 (2013). DOI: 10.1126/science.1228282 
1.	African
2.	Amazonian
3.	Arctico-Siberian
4.	Australian
5.	Chinese
6.	Eurasian
7.	Guineo-Congolian
8.	Indo-Malayan
9.	Japanese
10.	Madagascan
11.	Mexican
12.	North American
13.	Novozelandic
14.	Oriental
15.	Panamanian
16.	Papua-Melanesian
17.	Polynesian
18.	Saharo-Arabian
19.	South American 
20.	Tibetan

Q26. When did you start working on bats?

Q27. What is your gender?  
1.	Male
2.	Female
3.	Prefer not to answer

Q28. What is your date of birth?


Thanks. If you want to get informed about the results of the survey, do not hesitate to leave us your email address in the box below or to drop us a line otherwise just leave the box empty and click on the Continue button.
