# Supplementary material for: Bats and Academics: How Do Scientists Perceive Their Object of Study?
Source: PLoS One. 2016 Nov 10;11(11):e0165969. doi: 10.1371/journal.pone.0165969 (PMC5104368; doi:10.1371/journal.pone.0165969)
Supplement: S2 File — (RTF) [file pone.0165969.s005.rtf]

S2 File. Combination of keywords used to select participants in the survey.


TI = (Chiroptera OR bats OR Bat) 
NOT TS=(SWIFT/BAT) 
NOT TS=(Burst Alert Telescope)
NOT TS=(brown adipose tissue) 
NOT TS=(Dendropsophus) 
NOT TS=(bat-eared fox) 
NOT TS=(Bermuda Atlantic Time Series) 
NOT TS=(BAT-R-US) 
NOT TS=(baseball) 
NOT TS=(cricket) 

This search was repeated for every year between 2010 and 2014 with the coding PY=(Year of interest).
