# Supplementary material for: Bats and Academics: How Do Scientists Perceive Their Object of Study?
Source: PLoS One. 2016 Nov 10;11(11):e0165969. doi: 10.1371/journal.pone.0165969 (PMC5104368; doi:10.1371/journal.pone.0165969)
Supplement: S3 File — (RTF) [file pone.0165969.s006.rtf]

S3 File. Statistical analysis of the factors explaining the cause of the global decline of bats according to responders.


Statistical analysis of the ranking of deforestation as a cause of the global decline of bats


                    Estimate  Std. Error t value  Pr(>|t|)    
(Intercept)         2.290882   0.142395  16.088  < 2e-16 ***
Theoretical        -0.212470   0.122574  -1.733  0.08356 .  
Taxonomy           -0.152722   0.101027  -1.512  0.13116    
Conservation        0.157273   0.084156   1.869  0.06215 .  
WorkBatInfection    0.174986   0.094966   1.843  0.06590 .  
PercentageBats     -0.002211   0.001330  -1.662  0.09704 .  
PercDesk           -0.002549   0.001564  -1.630  0.10366    
ManipulateAlivebat -0.360660   0.098144  -3.675  0.00026 ***
SamplesAliveBats   -0.179661   0.095416  -1.883  0.06021 .  


Statistical analysis of the ranking of intensive agriculture as a cause of the global decline of bats


                    Estimate  Std. Error t value  Pr(>|t|)    
(Intercept)         2.692697   0.121925  22.085  < 2e-16 ***
Conservation       -0.328102   0.107093  -3.064 0.002287 ** 
PercLab             0.008892   0.002606   3.413 0.000688 ***
ManipulateAlivebat  0.370165   0.117961   3.138 0.001787 ** 
SamplesAliveBats   -0.256847   0.115959  -2.215 0.027148 *  


Statistical analysis of the ranking of urbanization as a cause of the global decline of bats


                  Estimate  Std. Error t value  Pr(>|t|)    
(Intercept)       3.702351   0.196730  18.819  < 2e-16 ***
Conservation      0.307184   0.129221   2.377  0.01777 *  
WorkBatInfection -0.355847   0.140767  -2.528  0.01174 *  
PercentageBats   -0.005659   0.001916  -2.954  0.00327 ** 
PercDesk          0.004535   0.002349   1.931  0.05402 .  


Statistical analysis of the ranking of climate change as a cause of the global decline of bats

                  Estimate  Std. Error t value  Pr(>|t|)    
(Intercept)       3.247243   0.136766  23.743   <2e-16 ***
Theoretical       0.242894   0.181559   1.338   0.1815    
PercentageBats    0.003507   0.001853   1.893   0.0589 .  
PercLab          -0.004569   0.002924  -1.563   0.1187    
SamplesAliveBats  0.298366   0.131196   2.274   0.0233 *  


Statistical analysis of the ranking of infectious diseases as a cause of the global decline of bats


                 Estimate  Std. Error t value  Pr(>|t|)    
(Intercept)        3.8172     0.1080  35.333  < 2e-16 ***
Physiology        -0.4549     0.1944  -2.340  0.01964 *  
Taxonomy           0.1993     0.1549   1.287  0.19877    
Parasitology       0.2492     0.1458   1.709  0.08790 .  
SamplesAliveBats   0.3514     0.1308   2.686  0.00744 ** 


Statistical analysis of the ranking of hunting as a cause of the global decline of bats


            Estimate  Std. Error t value  Pr(>|t|)    
(Intercept)  5.17008    0.05637  91.720   <2e-16 ***
PopGen      -0.26099    0.13726  -1.901   0.0577 .  
