# Supplementary material for: Bats and Academics: How Do Scientists Perceive Their Object of Study?
Source: PLoS One. 2016 Nov 10;11(11):e0165969. doi: 10.1371/journal.pone.0165969 (PMC5104368; doi:10.1371/journal.pone.0165969)
Supplement: S4 File — (DOC) [file pone.0165969.s007.doc]

Multiple Component Analysis on the use of protection tools while manipulating alive bats for researchers working on bats infections.

Multiple Component Analysis on the use of protection tools while manipulating alive bats for researchers not working on bats infections.

Multiple Component Analysis on the use of protection tools while manipulating samples from alive bats for researchers working on bats infections.

Multiple Component Analysis on the use of protection tools while manipulating samples from alive bats for researchers not working on bats infections.
